# Improved EMAT Sensor Design for Enhanced Ultrasonic Signal Detection in Steel Wire Ropes

**DOI:** 10.3390/s24227114

**Published:** 2024-11-05

**Authors:** Immanuel Rossteutscher, Oliver Blaschke, Florian Dötzer, Thorsten Uphues, Klaus Stefan Drese

**Affiliations:** 1 Institute for Sensor and Actuator Technology, Coburg University of Applied Sciences and Arts, Am Hofbräuhaus 1B, 96450 Coburg, Germany; oliver.blaschke@hs-coburg.de (O.B.); thorsten.uphues@hs-coburg.de (T.U.); klaus.drese@hs-coburg.de (K.S.D.); 2 Department of Mechanical Engineering, Technische Universität Ilmenau, Ehrenbergstraße 29, 98693 Ilmenau, Germany

**Keywords:** electromagnetic acoustic transducers, EMAT, ultrasonic, steel rope, wire breaks, neural network, transformer, pre-training

## Abstract

This study is focused on optimizing electromagnetic acoustic transducer (EMAT) sensors for enhanced ultrasonic guided wave signal generation in steel cables using CAD and modern manufacturing to enable contactless ultrasonic signal transmission and reception. A lab test rig with advanced measurement and data processing was set up to test the sensors’ ability to detect cable damage, like wire breaks and abrasion, while also examining the effect of potential disruptors such as rope soiling. Machine learning algorithms were applied to improve the damage detection accuracy, leading to significant advancements in magnetostrictive measurement methods and providing a new standard for future development in this area. The use of the Vision Transformer Masked Autoencoder Architecture (ViTMAE) and generative pre-training has shown that reliable damage detection is possible despite the considerable signal fluctuations caused by rope movement.

## 1. Introduction

Measuring the operational stability of steel wire ropes is crucial for retaining human and process safety. In contradiction to chains where the whole system fails if a single chain link breaks, faults in the wires of a steel cable are less critical as the adjacent wires can easily absorb the load. If a single wire within a wire rope breaks, the rope’s breaking strength is usually only minimally reduced, and this reduction typically affects only a short section of the rope. Consequently, a wire rope can remain fully functional despite the breakage of individual strands provided that these breaks are evenly distributed [1].

The key criterion for determining whether a wire rope has reached its maximum allowable number of wire breaks is not the total number of breaks but rather the number of breaks per defined length of the rope. This criterion is specified in various national and international standards, such as the ISO 4309 norm [2]. The permissible number of wire breaks within a rope section is defined as a multiple of the rope’s diameter. Therefore, it is possible for a crane rope with 10,000 evenly distributed wire breaks to remain in service while another rope with only 15 wire breaks concentrated over a few centimetres must be replaced. The accurate detection of these breaks is crucial to ensure the safety of conveyor systems, ropeways, and lifts.

In a recent review regarding different kinds of damage to steel wire ropes and their detection, Mazurek [3] provided a comprehensive overview on the state-of-the-art sensor solutions. The methods can be grouped into subclasses utilising either magnetic fields, thermal imaging, radiography, acoustic emissions, or ultrasonic guided waves. Starting with the magnetic field options, the magnetic flux method is a prominent approach where either a permanent or alternating magnetic field in combination with a magnetic flux sensor, e.g., a Hall sensor, scans the steel rope for damage [4,5,6]. If a defect is present, the magnetic field is subject to variations that are detectable to the sensor, thus eventually providing a quantitative estimate regarding the damage of the rope. While this method can provide information about a vast area of damage types (wire breaks, wear, and corrosion) and the ability to measure online, Mazurek [3] points out the long measurement time, which can be a drawback depending on the use case. Another approach uses eddy currents induced by the magnetic fields in the steel rope, which are disturbed in the vicinity of defects [7]. It is described that this technology provides fast results even in conditions with high temperatures, but it can mostly detect defects on the surface of the ropes, and there remain some problems involving skin effects that have to be solved [3]. Another method involves thermal imaging, where the rope segments are heated by induction and the defects on the surface are identified by an infrared camera [8,9]. In the literature, it is stated that this approach can provide information about the rope’s condition even with arbitrary rope materials and properties as well as different cross-sections [3]. On the other hand, the handling of the different temperatures of the sample and its surroundings is rather prone to errors in work site conditions in contrast to laboratory tests [10]. Radiography may also be one of the relevant methods. This approach mainly focuses on the absorption and penetration of gamma rays or X-rays as they were emitted on the surface of the rope and measured on the backside [11]. It was found that it can provide reliable results but comes at a relatively high cost and may be harmful to health and the environment [3]. There are also techniques involving ultrasound. These can be grouped into acoustic emission and ultrasonic guided wave methods. The first examine the backscattered acoustic signal of a defect within the wire rope and can measure a wide range of different defects but can be unreliable due to usually low signal-to-noise ratios [12,13,14]. The latter use ultrasonic transducers with direct contact to the rope diameter and induce, typically through piezoelectric effects, a mechanical wave in the ultrasonic frequency band. This wave propagates through the wires of the rope and interacts with the defects. The measured signal contains the information about the amount and location of the damage [15,16,17]. While this technique can provide insights about the defects in the inner layers of the rope, the sensors usually require physical contact in relation to the sample. Considering the uneven circumferential geometry of the steel wire rope, this makes an online measurement with moving rope systems difficult.

A promising approach to mitigate the drawbacks of ultrasonic guided wave sensors can be the contactless generation of the ultrasonic waves in ferromagnetic steel wire ropes through magnetostriction. With electromagnetic acoustic transducers (EMATs) in a symmetrical configuration around the steel rope, it is possible to excite symmetric ultrasonic modes in the wires of the steel rope [18]. These modes interact with the damaged parts of the rope and can thus indicate if a rope has to be replaced. This technique is also able to provide full coverage of the cross-section of the rope [19].

The EMAT approach can offer two working principles in rope damage detection. On the one hand, a back-reflected acoustic signal from the damage position can be evaluated, which enables analysing the number and position of wire breaks [18,19,20,21,22,23], assuming that multiple reflection signals do not superimpose and are thus differentiable. On the other hand, a transmission configuration where the rope segment between the transmitter and the receiver EMAT is evaluated is possible [24]. While the first approach can be viewed as an active defect detection method, the latter can be described as a passive detection approach because no reflection signal is evaluated and the information about the defects lies in the direct acoustic signal.

For online monitoring of steel wire ropes, passive defect detection can be more suitable to measure the operational stability rather than active detection because, in most cases, only the amount of defects at a given length interval are relevant. Also, multiple defects in a small rope section are less problematic to this evaluation scheme as no reflection signals have to be differentiated.

Therefore, we propose a transmission EMAT sensor system with a dedicated transmitter and receiver to generate ultrasonic guided waves in a contactless manner, which enables the analysis of steel wire ropes for common defects like wire breaks and abrasion. We analysed different evaluation schemes from traditional signal processing techniques to artificial neural network models on the basis of convolutional neural networks (CNNs) as these are capable of handling temporal series data that are typical of signal evaluation [25,26,27]. We incorporated a method where we can also add scalar data through dense layers to account for the characteristics found in the signals. While these models can predict the kind and degree of rope damage even in the presence of interference variables like soiling, the wire rope has to be static during the measurement since the signals experience significant fluctuations on a moving rope. Measuring a moving rope has major advantages in terms of processing time and enables continuous monitoring. We propose another evaluation scheme for these use cases. We found that a transformer-based deep learning neural network relying on positional encoding and self-attention mechanisms [28] can differentiate intact from damaged rope segments even in dynamic scenarios. Additionally, through generative pre-training [29,30] using large quantities of synthetically generated ultrasound signals, it was possible to increase the accuracy to over 99%.

## 2. Materials and Methods

### 2.1. Theoretical Background

Magnetostriction is a phenomenon where certain ferromagnetic materials change their shape or dimensions in response to a magnetic field [31,32]. This property is utilised in the operation of electromagnetic acoustic transducers (EMATs), which are devices that generate and receive ultrasonic waves for non-destructive testing applications. When a magnetic field is applied to a ferromagnetic material, the alignment of magnetic domains within the material changes. This realignment causes a strain in the material, leading to a change in its dimensions. The strain generated can be compressive or tensile depending on the nature of the magnetic field and the material properties. This dimensional change is the core principle behind magnetostriction.

In the context of EMATs, an alternating magnetic field is used to induce dynamic magnetostrictive strains in the material. These strains generate mechanical vibrations, which propagate as ultrasonic waves [33]. The EMAT consists of a coil to generate the dynamic magnetic field and permanent magnets to bias the material, ensuring efficient generation of ultrasonic waves.

**Figure 1:**
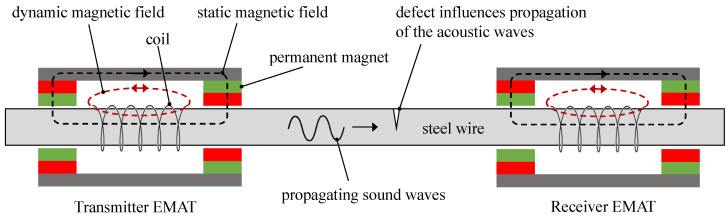
Schematic illustration of the generation of ultrasonic signals in steel cables by means of magnetostriction. The sound waves generated by the transmitter are influenced by defects in the steel cable. The sound waves are then detected by the receiver.

The measurement setup for detecting wire breaks in steel cables using electromagnetic acoustic transducers (EMATs) involves the interaction of magnetic fields and ultrasound waves, as shown in Figure 1. This setup consists of two primary components: a transmitter EMAT and a receiver EMAT. The transmitter EMAT initiates the process by using a coil connected to an AC source to generate a dynamic magnetic field. This field interacts with the steel wire, inducing magnetostrictive vibrations within the material. Surrounding the coil, permanent magnets establish a static magnetic field, which biases the steel cable to optimise the magnetostrictive effect. As a result, the combined action of the static and dynamic magnetic fields produces mechanical vibrations that generate acoustic waves.

As these acoustic waves travel along the steel cable towards the receiver EMAT, their propagation characteristics are directly influenced by the wire’s physical condition in between transmitter and receiver. Structural anomalies, such as wire breaks or other defects, influence the wave propagation, altering the amplitude, phase, or time-of-flight of the acoustic waves. Structurally similar to the transmitter, the receiver EMAT consists of a coil and permanent magnets designed to detect these incoming acoustic waves.

One of the significant advantages of using EMATs for this purpose is their ability to perform non-contact inspections. Unlike traditional methods that require direct contact with the material under inspection, EMATs can detect defects in moving ropes without the need for a coupling medium. This capability is particularly beneficial for inspecting ropes in dynamic environments, such as those found in cranes or elevators, where the ropes are constantly in motion. Furthermore, EMATs are versatile and can operate effectively under harsh conditions, including high temperatures and rough surfaces. This versatility enhances their applicability in various industrial settings.

Since the EMATs utilise acoustic guided waves in the ultrasonic frequency band, there are some considerations on this topic. As the steel ropes are assembled from single wires, these can be viewed as waveguides. For approximation, the single wires are treated as infinitely long, straight cylinders. This assumption is valid for small lay angles below 7.5° as the dispersion characteristics are only slightly affected in this area [34]. The ropes in this work fulfill this criterion.

One has to distinguish between different mode shapes that can be present in cylindrical guided waves [35]. There are longitudinal modes L(m,n) with a symmetric appearance around the centreline of the cylinder and displacement in axial and radial directions. On the other hand, there also exist torsional modes T(m,n) with a displacement in circumferential direction. Lastly, flexural modes F(m,n) have an asymmetric shape around the centreline and displacements in all three directions. The index *m* indicates the number of circumferential wavelengths and *n* the mode number in radial direction. With increasing frequency, higher-order modes appear. In this work, only the fundamental modes are considered.

**Figure 2:**
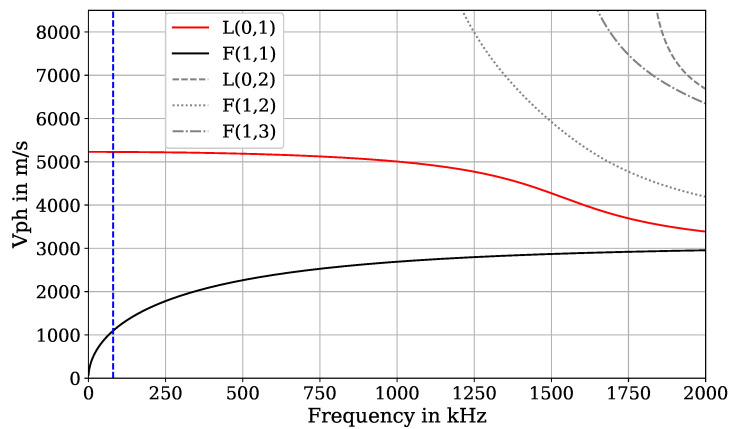
Dispersion diagram of the phase velocity of different modes for a steel wire with similar dimensions as the experimental samples. Black curve: F(1,1) fundamental mode. Red curve: L(0,1) fundamental mode. Grey curves: higher-order *F* and *L* modes. Blue dashed line: excitation frequency of this study at 80 kHz.

While *L* modes and *F* modes are dispersive, indicating that the group velocities and phase velocities are frequency-dependent, the velocities of *T* modes remain constant regardless of the frequency. Since *T* modes are rather difficult to excite with a symmetric coil EMAT, the L(0,1) mode was used in this work. In Figure 2, it can be seen that the L(0,1) mode has only a low-dispersive characteristic in the frequency range up to 150 kHz, which is beneficial for signal evaluation techniques as the shape of the waveform remains nearly constant during propagation. It is also advantageous that the higher-order *F* and *L* modes are not present in this frequency range, which simplifies the evaluation. This feature and the fact that the L(0,1) mode has the highest group velocity enable applying different evaluation schemes. An excitation frequency of 80 kHz was employed as this represented an optimal compromise between sufficient signal amplitude and therefore signal-to-noise ratio and the ability to separate the ultrasonic transmission signal from the inherent electromagnetic crosstalk between the sending and receiving EMAT.

The dispersion diagram shows the phase velocity for a given frequency for which it is important to determine the wavelength of the excited signal and thus the design of the EMAT since the coil widths can be adapted to the wavelength of the L(0,1) mode for a pure mode excitation and thus reduce the contributions of other disruptive modes in the recorded signal. This design process is described in more detail in the following section.

### 2.2. EMAT Design

To identify a suitable initial design for subsequent experimental optimisation, numerical simulations were performed using the magnetic fields module (mf) in COMSOL Multiphysics. The simulation was focused on the design of the static magnetic bias field. The goal is to provide a homogeneous magnetic field of defined magnitude inside the segment of the steel wire, where the coils generate the alternating magnetic field. The presence of the static magnetic field is crucial for biasing the material, thereby facilitating the efficient generation of ultrasonic waves through magnetostriction.

**Figure 3:**
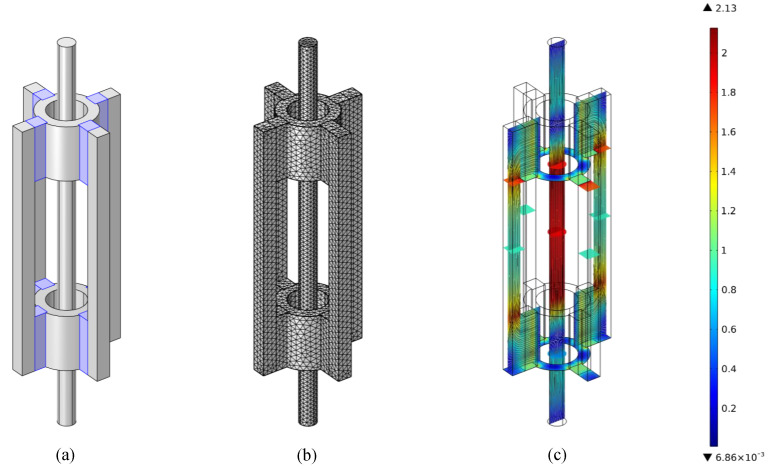
Example of a simulated configuration composed of the steel wire, surrounded by the pole shoes, permanent magnets, and iron yokes (**a**), the mesh used for the simulations (**b**), and the resulting magnetic flux density in Tesla (**c**).

As illustrated in Figure 3, pairs of permanent magnets at the ends of the EMAT are used as the source of the static bias field. Pole shoes guide the field from the magnets to the steel wire. The wire constitutes the inner path of the magnetic circuit, whereby the magnetic field is predominantly aligned parallel to the wire. To efficiently close the magnetic circuit on the outside, iron yokes are placed parallel to the wire, attached to the backside of the magnet pairs. Although not considered in the simulations, the coils will be located within the surrounding iron yokes and are wound around the steel wire to produce a magnetic field that is predominantly aligned parallel to the wire and the bias field too.

All components in the simulation are modelled by Ampere’s law with different magnetisation models and parameters according to the different materials. The properties of the magnets, steel wire, iron yokes, pole shoes, and the surrounding air were taken either directly from COMSOL‘s material library or configured according to available data sheets and publications. Specifically, the permanent magnets are modelled by a remanent flux density magnetisation model. The remanent flux density norm is set to $\left|\right|B_{r}\left|\right|=1.3\;T$ and the recoil permeability to $\mu_{rec}=1.05$ as commonly found in Neodymium magnets. The direction of the remanent flux density faces radially inwards and outwards for the upper and lower magnets, respectively. The remaining properties are approximated by the Nonlinear Permanent Magnet material model from the COMSOL library. The pole shoes, yokes and wire are modelled by a B−H curve magnetisation model and the material properties are derived directly from the material library. The Soft Iron (With Losses) material is used for the pole shoes and yokes, while the steel wire is modelled by the Low Carbon Steel 1008 material. The surrounding air is modelled by a relative permeability magnetisation model with parameters taken from the material air in the library. Deviations from the actual material properties are tolerable here because the simulations only serve as a semiquantitative aid for identifying coherences and finding a suitable initial design that is eventually optimised experimentally in any case.

During all simulations, the free length between the magnet pairs was kept fixed to 100 mm such that three coil segments, each with a length of λ/2, would fit between them. The inner diameter of the pole shoe was fixed to 27 mm, such that commonly used Teflon bushings could be inserted to center the steel wire. For the investigated wire with a diameter of 12 mm, this yields an air (or Teflon) gap of 2.5 mm. Permanent magnets with a cross-section of 10 mm × 10 mm and a length of 40 mm and a direction of magnetisation along one of the short sides are used because of their common availability.

Remaining parameters of the geometry and configuration have been varied in the simulations to study their impact on the magnetic field inside the steel wire. The number of magnet pairs and therefore the total cross-sectional area of the magnets turned out to have the strongest impact on the magnetic flux density inside the steel wire. Stacking magnets on each other and thereby increasing the thickness of the magnets along their direction of magnetisation had a smaller effect in comparison. The iron yokes only had a minor impact as long as their cross-section was large enough for them to not be saturated. A higher thickness of the pole shoe had a benefit for the homogenisation of the flux density, especially for a low number of magnet pairs, but slightly decreased the field‘s magnitude. If only one pair of magnets is used, the lack of symmetry poses much higher requirements on the centering of the steel wire as the flux density inside the wire will depend on its lateral position more strongly than for symmetric designs.

**Figure 4:**
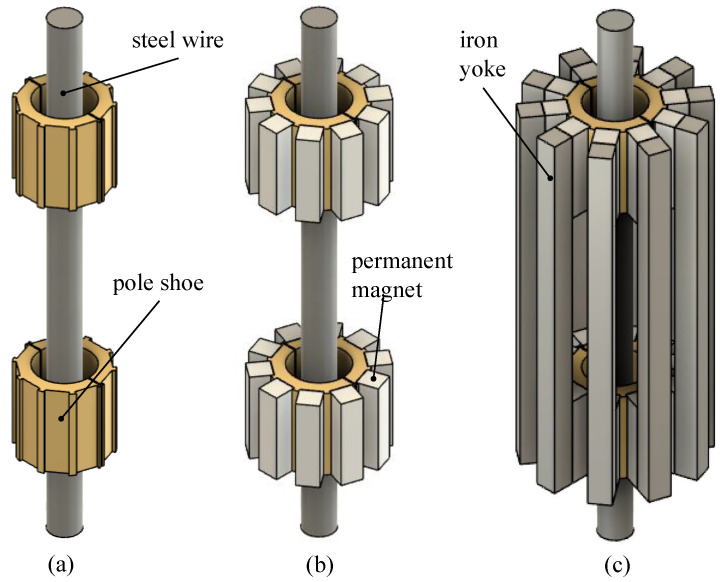
Setup with the steel wire encircled by pole shoes (**a**). Addition of permanent magnets directly on the pole shoes (**b**). Addition of iron yokes to help complete the magnetic circuit (**c**).

The design illustrated in Figure 4 is based on the results and findings from these simulations. The pole shoes are thick enough to sufficiently homogenise the field from the magnets. They can accommodate up to ten magnets each, and the number of magnet pairs can be varied from zero to ten to enable adjustments of the magnetic bias field’s magnitude in the experimental investigations. This enables a systematic investigation of signal strengths in relation to the magnitude of the permanent magnetic field, as investigated in Section 2.4. The iron yokes feature a cross-section of 10 mm × 10 mm, which suffices to avoid saturation because the sum of their cross-sections is equal to or larger than that of the steel wire.

In the following section, the detailed construction of the derived EMAT sensors is described. This includes an in-depth look at the components and their assembly. Figure 4 illustrates the detailed construction of an EMAT sensor, focusing on the components responsible for generating the permanent magnetic field. At the center of the setup is the steel wire, around which the EMAT sensor components are arranged. The permanent magnets are placed directly on the pole shoes composed of steel, and the iron yokes complete the assembly of the static magnetic circuit.

**Figure 5:**
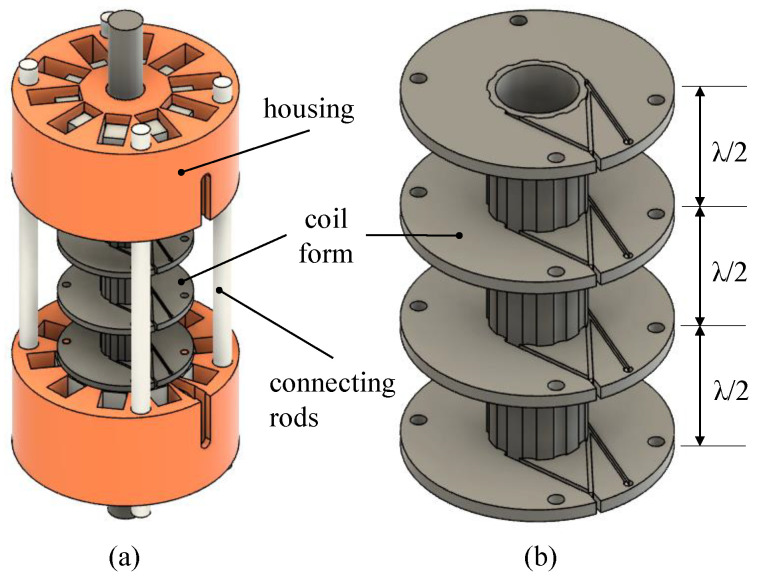
Assembly of the EMAT sensor showing the housing that encloses the components, connecting rods that maintain alignment, and the coil form (**a**). Detailed view of the coil form, highlighting the three segments, each corresponding to half the wavelength (λ/2) of the ultrasonic waves (**b**).

To generate the dynamic magnetic field, a special coil body was designed, as shown in Figure 5. The coil body, located in the center of the EMAT sensors, was divided into three segments, each segment corresponding to half the wavelength (λ/2) of the ultrasonic waves. Adjacent segments of the coil were wound in opposite directions using copper wire. The counter-winding had the effect that, depending on the winding direction, the bias magnetic field was either amplified or attenuated. This made it possible to excite sound waves with a specific wavelength in a more frequency-specific way. Housing parts and connecting rods secured the magnets and iron yokes. Connecting rods provided the EMAT the necessary stability for moving ropes and harsh environments.

**Figure 6:**
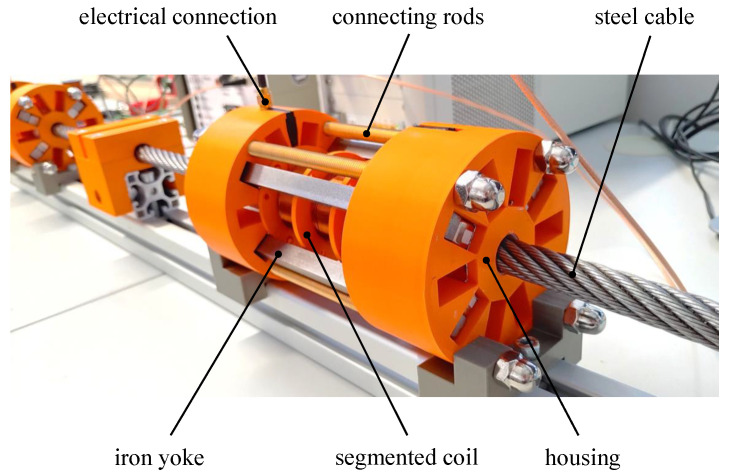
Photo of a fully assembled EMAT sensor for generating sound waves in steel cables. Neighbouring segments of the coil are wound in opposite directions to excite signals with a defined wavelength.

Figure 6 shows a fully assembled EMAT sensor that is constructed with the following configuration: It includes 8 permanent magnets, forming 4 magnet pairs, which are interconnected by 4 iron yokes. The coil consists of three segments, each with n=500 windings per coil segment (λ/2). The connecting rods were composed of brass to avoid interference with the magnetic field, providing structural stability. The housing components and the coil body were produced using 3D printing. In order to minimise friction and abrasion between the sensor and the cable, a replaceable Teflon cylinder was located inside the EMAT.

### 2.3. Preparation and Testing

**Figure 7:**
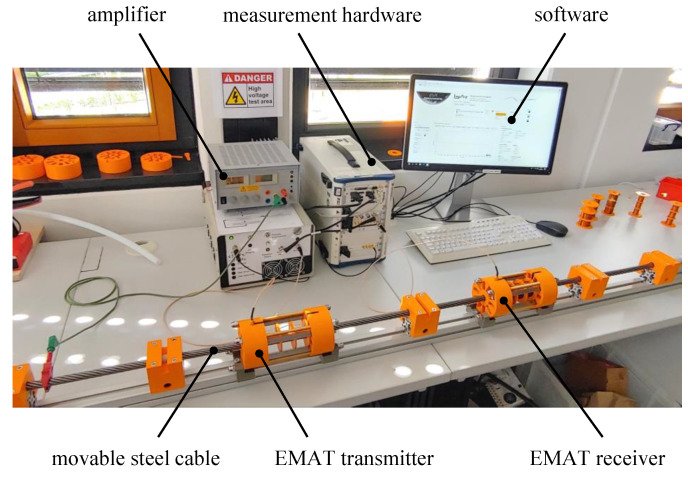
Photo of the test rig designed for performing damage detection tests on steel cables.

The test rig shown in Figure 7 is designed for the evaluation of the performance of electromagnetic acoustic transducers. It comprises an EMAT transmitter and receiver, spaced 40 cm apart, ensuring a consistent propagation path of ultrasonic signals along the cable. The steel cable ran through these sensors and was shifted to systematically test various sections. The rig is equipped with National Instruments measuring hardware, including a 14-bit signal generator (NI PXI-5412) and a 12-bit oscilloscope (NI PXI-5105), capable of sampling rates up to 60 MHz. The excitation signals generated by the signal generator were amplified up to 100 Vpp using a self-developed amplifier. Custom-developed software supported the configuration of measurement hardware, as well as the automated operation of experiments and the evaluation and storage of measurement data.

### 2.4. EMAT Optimisation

Due to the inherently low efficiency of magnetostrictive energy conversion from magnetic to acoustic energy, EMAT sensors require optimisation to effectively detect faults in steel cables using ultrasound. The primary goal was to excite ultrasonic waves with the highest possible amplitude in steel cables, as outlined in Section 2.1 and illustrated in Figure 1. A robust signal with a beneficial signal-to-noise ratio and reduced sensitivity to attenuation requires high amplitudes. The optimisation was performed for the 12 mm diameter steel cables mentioned in Section 2.5. Cables with different diameters or composed of other materials may require adjustments to the EMAT configuration.

To achieve this, two main parameters were adjusted: the strength of the static magnetic field and the dynamic magnetic field’s effectiveness, which is influenced by the inductance of the coil. The static magnetic field strength is controlled by varying the number of permanent magnets within the sensor setup. This field requires being strong enough to induce significant magnetostriction below the magnetic saturation limit of the steel cable. Exceeding the saturation threshold can decrease the efficiency of the sensors. If the magnetic field is too strong, it forces all the magnetic domains into alignment, stabilizing the material’s magnetic state and making it less sensitive to further magnetic field changes. This results in diminished effectiveness of the coil’s magnetic field modulation, leading to weaker generation of ultrasound and reducing the sensor’s overall effectiveness.

In addition to optimizing the static magnetic field, adjusting the dynamic magnetic field is required for generating ultrasonic waves with sufficiently high amplitudes. The effectiveness of this field is primarily governed by the inductance of the coil, which is influenced by the number of windings. More windings result in a larger coil inductance, which enhances the coil’s ability to generate a higher voltage output in response to the magnetic fields induced by the returning ultrasonic waves in the case of the receiver.

In the case of the transmitter, it was shown that a coil design with relatively few windings leads to better efficiency. Although this phenomenon was not analysed in detail, the reason for this could be that fewer windings result in lower impedance, enabling a higher current when a given voltage is applied. This higher current leads to a strong magnetic field while maintaining lower energy losses typically associated with higher impedance. Additionally, higher inductance associated with more windings can lead to increased heat generation, which may further reduce current flow.

**Figure 8:**
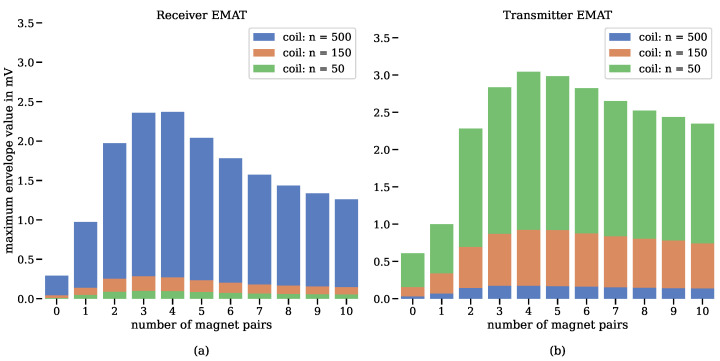
Impact of varying the number of magnet pairs and coil windings on the signal strength of ultrasonic signals for the receiver (**a**) and transmitter (**b**).

Figure 8 illustrates the relationship between the signal strength of the ultrasonic signals and the number of magnet pairs, as well as the number of coil windings per coil segment, for the receiver (a) and the transmitter (b). A total of 11 different magnet pairs were analysed, ranging from 0 (indicating no static magnetic field) to 10 magnet pairs, which equals 20 permanent magnets per EMAT for a very strong permanent magnetic field. It was observed that four symmetrically arranged magnet pairs produce the highest signal strength for both the receiver and the transmitter.

The segmented coils were manually wound, with significantly different numbers of windings per coil segment (50, 150, and 500). The findings show that, in the context of the dynamic magnetic field, the receiver performed best with 500 windings per segment, while the transmitter achieved optimal results with 50 windings per segment.

**Figure 9:**
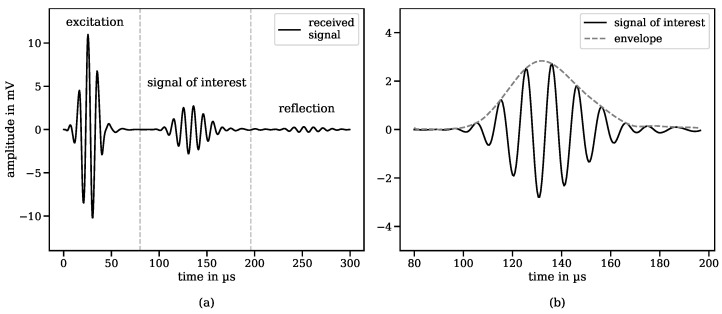
Received signal consisting of the electromagnetic crosstalk of the excitation signal, the actual signal of interest, and a reflected signal from the end of the cable (**a**). Signal of interest (detailed view) detected by the receiving EMAT, which is used to analyse rope damage (**b**).

Figure 9 displays the digitised received signal when both the transmitter and receiver are optimised with respect to the dynamic and static magnetic fields. The excitation signal at 80 kHz was amplified to 80 Vpp, resulting in a received signal amplitude of just over 3 mV.

### 2.5. Material Preparation

In this work, there were two common types of steel wire ropes covered. At first, a bare steel wire rope was used with a strand structure of 6 × 25 and a diameter of 12 mm. Second, a galvanised rope with a structure of 6 × 19 and diameter of 12 mm was analysed. Both ropes are in accordance with EN 12385-4 [36].

The main focus was on analysing defects on the ropes, especially broken wires and rope abrasion. Further, it was investigated if increased rope soiling can affect the significance of the measurement. For the preparation of the ropes, a Dremel tool was used to cut wire strand packages of three to four wires iteratively. Attention was paid such that, with every cutting process, a new wire strand was cut through. For the abrasion testing, an iterative filing process was conducted. The time for the filing was constant for every iteration. The soiling effect was simulated by treating the ropes with grease with a layer thickness between 5 mm and 10 mm.

### 2.6. Data Processing

Two different approaches were pursued for the processing of the time signals. On the one hand, a static method was examined that requires the rope to stand still for the measurements. This method features an artificial neural network where series-like data are processed through convolutional layers, and simultaneously parallel scalar data features from the measurements were computed in a dense network as well. Both processing strands merge together at the end to provide either predictions about the kind of rope defect or the amount of damage based on the defect scenario. On the other hand, a transformer-based neural network was constructed that also enables measurements on moving ropes for a dynamic use case. We tested four different model sizes based on the Vision Transformer Masked Autoencoder Architecture (ViTMAE) [30]. In addition, the influence of generative pre-training was investigated to further increase model accuracy.

For better readability, both processes are depicted in detail in their associated subsections in Section 3.

## 3. Results

### 3.1. Static Case

#### 3.1.1. Conventional Signal Evaluation Strategies

At first, conventional signal evaluation techniques were applied to the measured time signals. This included time domain evaluation, like the maximum of the envelope of the signal, the positions of zero crossings, and measuring the time of flight by calculating a cross-correlation between the sent and received signals. Further, a spectral analysis was conducted by applying a fast Fourier transformation with the FFT algorithm. In the frequency spectrum, the maximum and mean of the amplitude as well as the centre frequency of the spectrum were analysed.

**Figure 10:**
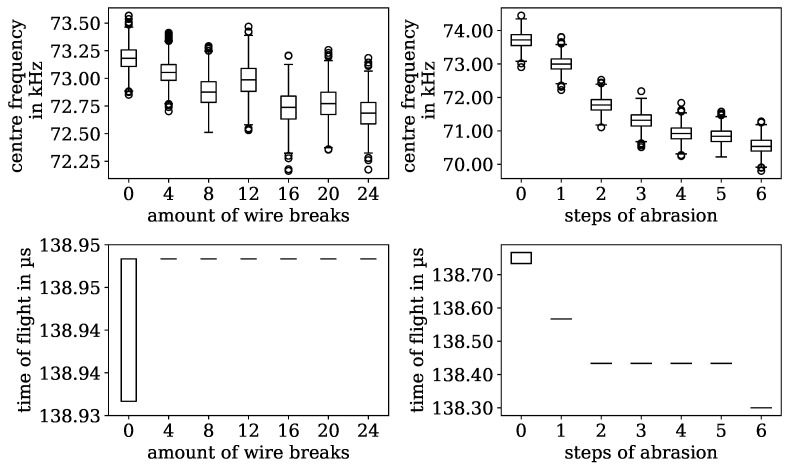
Different measures for rope damage detection as boxplot analysis. (**Upper row**) centre frequency against either wire breaks or abrasion levels. (**Lower row**) time of flight against either wire breaks or abrasion levels.

After the initial screening of the promising influencing factors for the measurement, a principal component analysis (PCA) was conducted to construct the most impactful components out of the features and hence limit the dimension of the information system to two for visualising the results. The goal was also to identify the most informative features for the latter neural network approach. The most significant measures were the centre frequency of the amplitude spectra of the Fourier-transformed time signal, which is shown in Figure 10, as well as the mean and maximum values of the amplitude. The standard deviation of the amplitude in the time domain was found to be another relevant measure. The time of flight value is very important to distinguish between wire breaks, where the value remains mostly constant, and rope abrasion, where a significant effect can be measured, as depicted in Figure 10.

**Figure 11:**
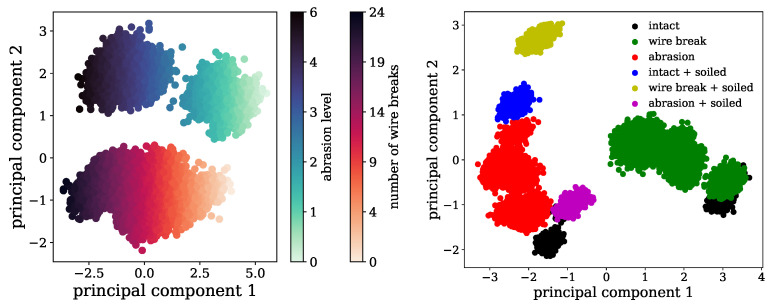
Principal components for intact and defective steel wire rope measurements. (**Left**) analysis of a blank steel rope with increasing number of wire breaks and abrasion levels. Principal component 1 relates to the progress of damage, while principal component 2 differentiates wire breaks from abrasion. (**Right**) analysis of a galvanised steel rope with increasing number of wire breaks, abrasion levels, and soiling effects.

The results of the PCA for the blank steel wire ropes are depicted in Figure 11 on the left, where the various input measures were recombined into two principal components, whereas component 1 takes up 48% and component 2 30% of the explained variance. Evidently, the wire breakage can be distinguished from the damage through abrasion as the different point clouds run parallel. The colour progress indicates that it was possible to distinguish the level of damage for both types as well. Noticeable is the gap in the abrasion data points, which might be produced through inconsistent filing and the fact that, in the beginning of the machining process, due to the circular cross-section of the ropes, a higher change in the circumference is achieved by constant filing time and applied force, which can lead to a nonlinear correlation.

Evaluating the PCA of the experiments with the galvanised rope in Figure 11 (right side), the data points of the wire break and abrasion datasets are again distinguishable. This time there are also data points for measurements with soiled rope segments available. These behave very different to the other datasets and do not fit well into the scope of the other experiments. Hence, it seems that, because of the soiling, a differentiation between the types of damage or damage levels cannot be guaranteed. Another important point is that the “intact” conditions for the two experiments with the different damage types are also shifted, which means that evaluating different rope segments leads to offsets in the data. This can be explained by fluctuations in the rope or wire diameter, the applied tension, or material irregularities.

Regarding the results of the PCA evaluations, we conclude that, while the data points can be lined up according to the damage level, it is not trivial to distinguish different damage types in steel wire ropes if the sensor will be used on different rope segments and soiling is present, at least with the conventional signal processing methods. Therefore, we checked if a deep learning neural network can distinguish the different kinds of conditions and also provide accurate predictions about the status of the ropes.

#### 3.1.2. Dense-Convolutional Parallel Networks

To differentiate between the various types of damage, we have created a classification network for both galvanised and bare ropes. For one part, the same scalar data as in the PCA were used as input data and processed with layers of fully connected neurons. In addition, the use of one-dimensional convolutional neural networks also allows series-type data to be processed and further nonlinear features to be extracted [25]. The input for the convolutional neural network was the cross-power spectrum density (CPSD) of the measured ultrasonic signal with the excitation signal. A fixed frequency range from 25 kHz to 150 kHz was used to limit and unify the sample size. The cross-power spectrum density is calculated as follows:

(1) $$CPSD=FFT{\left(excitation\right)}^{*}\cdot FFT\left(measured\right)$$

where FFT is the fast Fourier transformation of either the excitation or the measured signal and * denotes the complex conjugation. The convolutional layers and the fully connected layers with their related inputs are processed in parallel and merged at the end of the network through further layers of fully connected neurons. In the end, a triple output provided a classification estimator with the information if the rope was intact or damaged either by wire breaks or abrasion. The structure of the classifier neural network is presented in Appendix A.1. The classifier models were trained for 40 epochs at a constant learning rate of 0.001. To ensure that during the training no overfitting occurred, three dropout layers were introduced with a dropout rate of 0.2. For the positions of the dropout layers, refer to Appendix A.1. Additionally, a L2 regulariser with a bias of 0.01 was introduced at every CNN and dense layer at the parallel part of the network. The training was conducted on a dataset of approximately 33,000 measurements for the blank steel rope and the galvanised steel rope each. Each dataset was randomly split into 85% training data, 12.5% validation data during the training, and 2.5% test data for model testing.

**Figure 12:**
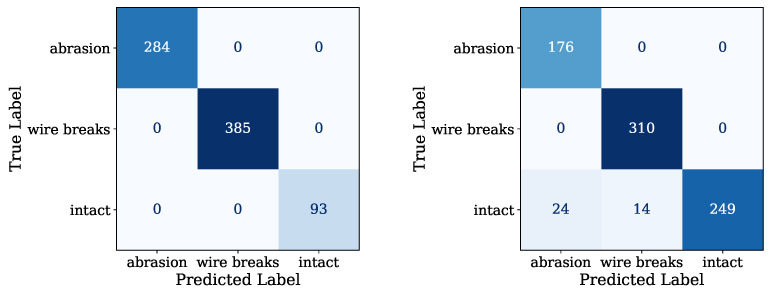
Confusion matrix of the classifier model for the bare steel wire rope (**left**) and for the galvanised steel wire rope (**right**).

For the final test evaluation, the predicted rope conditions of the models were plotted against the real conditions in a confusion matrix to ascertain the validity of the models. Looking at the map for the blank steel ropes in Figure 12 on the left side, the model predicted all three conditions without any errors. Noticeable is the fact that the data acquisition incorporated different measuring positions over the rope length to account for the fluctuations that were indicated by the PCA evaluations.

The confusion matrix for the galvanised steel rope model in Figure 12 on the right side reveals a similar result. Again, the majority of the rope conditions were estimated correctly. Only 2.8% of the measurements were predicted as false positive, whereas the most errors were predicted as wire breaks on an intact rope. Again, no false negative errors were found, which is favourable concerning security aspects. This was achieved by introducing weighting of certain conditions during the model training to tune the model to only providing false positive errors if any.

In the next step, the regressor models for the prediction of either the number of wire breaks or the abrasion levels for the galvanised steel wire ropes are presented. The structure and input data of these models were mostly the same as for the classifier models. Only the output layers were different to display a single value, which was, depending on the model, the estimate about the number of wire breaks or the amount of abrasion. The structure of the regressor model is also available in Appendix A.2. The training was performed on a dataset of 14,300 measurements for the wire break model and 7300 for the abrasion model. The splitting of the datasets was 85% training data, 12% validation data, and 3% test data. The dropout and L2 regulariser approach were the same as with the classifier models described prior. The training was conducted for 200 epochs with a learning rate of 0.001 for the wire break model and 100 epochs for the abrasion model with the same learning rate.

**Figure 13:**
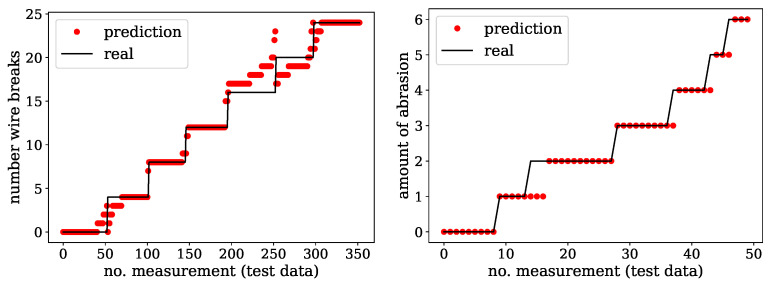
Predictions of the regressor models for the galvanised steel wire ropes on a test dataset. (**Left**) wire break predictions against real conditions. (**Right**) abrasion level predictions against real conditions.

The models were finally verified on the test dataset. Starting with the first one in Figure 13 on the left, the real number of wire breaks are plotted in ascending order against the model predictions. While most of the predictions agreed well with the real values, there was a range between 15 and 18 wire breaks where the model deviated. However, these differences were on a small scale and in a practical context; a deviation of one to three broken wires will most likely not be significant. The relative error for deviations larger than three broken wires was 9.6%. Why the deviations were mostly located in this range was not completely clear at this point. Most likely, the dataset for these wire break numbers was too small or the data were prone to errors.

The test of the abrasion model showed a similar result to the wire break prediction. Regarding Figure 13 on the right, most of the predictions were valid again. A few errors (10% as of the sample size) were determined, but the deviations are not more than a single level of abrasion, which seems to be acceptable. In conclusion, all the test runs, including those from both the classifier and regressor models, showed promising results. The neural network models successfully determined whether a steel wire rope, either bare or galvanised, was intact or damaged. It was also possible to provide information about the kind of damage and the degree of damage, even under conditions with additional impact like soiling.

### 3.2. Dynamic Case (Moving Cable)

#### 3.2.1. Conventional Signal Evaluation Strategies

The primary objective of the measurements on the stationary rope was to investigate whether a condition analysis is possible with the developed EMAT-based sensor system as well as to recognise the degree of damage. In practical applications, it is advantageous if the measurements are performed while the cable is moving. This capability would not only save significant time during the cable inspection but would also enable the system to act as a continuous monitoring tool. However, this approach comes with some challenges. While the received signal is very stable when the cable is stationary, there are strong fluctuations in the received signal when the cable is moving.

**Figure 14:**
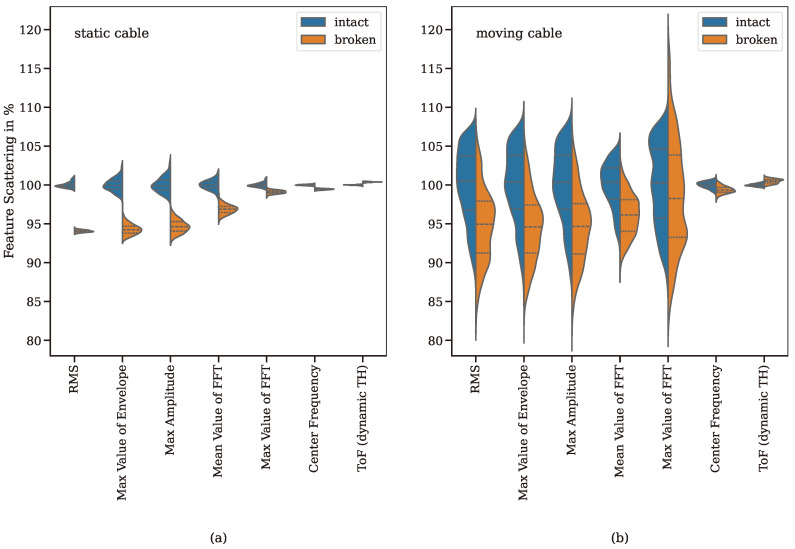
Comparison between the distributions of different features for a stationary cable (**a**) and a moving cable (**b**). While the features for the stationary cable are only slightly scattered, there is a significantly greater scatter of all features for the moving cable. As a result, it is no longer possible to distinguish clearly between defective and intact cable sections. The values are standardised in such a way that 100% corresponds to the mean value of the respective features of intact cable sections.

Figure 14 a shows that, in the case of an unmoved cable, a distinction can be made between defective and intact rope sections using simple features from the time signal or amplitude spectrum. In contrast, in the case of a moving cable, there is a scattering of the features so that a distinction based on individual features or a combination of several features is no longer possible (Figure 13b). This comparison only differentiates between intact and defective cable sections. A defective cable section means that 10 individual wires are broken over a length of 40 cm. This corresponds approximately to the discard criterion for the wire rope used in this setup. Due to the strong signal fluctuations, it can be concluded that analysing signals using conventional methods would not lead to reliable predictions. Therefore, as in the case of static ropes, an AI-based method is introduced in the following section.

#### 3.2.2. Transformer-Based Signal Processing

The conventional signal processing methods and dense/CNN-based networks achieved promising results in evaluating the conditions and levels of damage in static ropes. In the case of moving ropes, due to the strong fluctuations in the quality of the received signals and the resulting variance in the training data, a relatively new AI-based approach was investigated that enables pre-training using unlabelled ultrasonic signals. For this purpose, a transformer-based deep learning architecture based on positional encoding and self-attention mechanisms was used [28].

These mechanisms enable the transformer architecture to overcome the limitations of, e.g., convolutional neural networks (CNNs) and recurrent neural networks (RNNs). This includes processing sequential input data in parallel and recognising distant dependencies within a sequence from the first layer onwards [28]. This architecture, specially developed for the field of Natural Language Processing (NLP), has achieved ground-breaking success in this domain [37,38,39,40]. In addition, in the field of computer vision, it has been shown that, under certain conditions, established architectures can be outperformed by Vision Transformers (ViTs) [41].

The main challenge here is that transformers were able to achieve excellent results, particularly when a relatively large amount of labelled training data are available [41]. This is due to the low inductive bias compared to CNN- or RNN-based architectures, which allows the transformer model to recognise more complex patterns in the training data but at the cost of requiring a larger amount of labelled training data. To overcome this disadvantage and since the amount of labelled data is the limiting factor in many applications, the technique of generative pre-training can be used to improve the accuracy of a transformer model [41,42].

In this technique, the first step involves pre-training the model using a broad and extensive corpus of unlabelled data to build up a general understanding of the input data. The self-supervised task of reconstructing partially masked input data as accurately as possible is of major importance in this pre-training phase. This very general task does not only deepen the model’s understanding of the training data but also prevents the loss of important information, which is a common issue with task-specific predictions [30,37].

The second step is fine-tuning, where the pre-trained model is adapted to specific tasks by further training with available labelled data. This process allows the model to leverage the general understanding gained during pre-training, typically yielding better results than training solely from scratch with labelled data [30,37].

This technique is widely used in the field of NLP, known as masked language modelling, which improves model comprehension by predicting missing words in sentences [37,38,39,40]. Generative pre-training is also highly effective in computer vision, known as masked image modelling, where it enables models to learn robust features by predicting and reconstructing the masked sections of images, thereby enhancing their ability to perform complex tasks such as object detection [29,30,43].

Even though transformers are currently used successfully in more and more fields, the area of processing one-dimensional ultrasound raw signals is still largely unexplored. This study investigates whether the transformer architecture is applicable to processing ultrasonic raw signals to differentiate between defective and intact rope sections when the rope moves continuously during the measurement.

**Figure 15:**
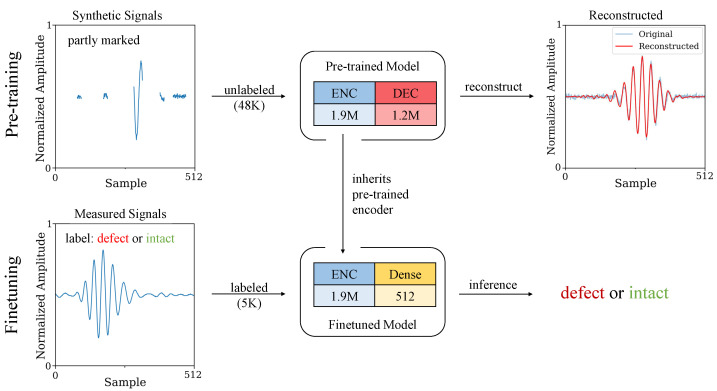
Principle of generative pre-training with subsequent fine-tuning using a ViTMAE optimised for ultrasonic signals. The transformer consists of two main components. Firstly, the large encoder to obtain a robust feature space for a general understanding of ultrasonic signals. Secondly, a smaller decoder that is used to reconstruct the masked signal components. After completing the pre-training, only the encoder is used for the downstream task. Either the complete downstream model can be fine-tuned (full fine-tuning) or only the final classification layer (dense) with just 512 parameters (linear probing).

For this purpose, the Vision Transformer Masked Autoencoder (ViTMAE) architecture [30] is used, which is an adaptation of the Vision Transformer (ViT) [41], designed specifically for self-supervised learning through masked image modelling. In the case of ultrasonic signals, the pre-processing procedure is adapted so that the model can process one-dimensional signals instead of images. Figure 15 shows the principle of generative pre-training and fine-tuning using a ViTMAE on the basis of ultrasonic signals. The dataset for the pre-training comprises 60,000 synthetically generated ultrasonic burst signals, which were split into training, validation, and test data in a ratio of 80/10/10.

The signals vary strongly in characteristics such as frequency, amplitude, number of periods, time of flight (ToF), and noise level. This diversity helps the model to acquire robust features and to build a comprehensive understanding of ultrasonic signals through the reconstruction of partially masked signal segments. For the subsequent fine-tuning, we used a labelled dataset from the moving rope that includes 6250 individual signals labelled as ‘intact’ and ‘defective’, which were also split into training, validation, and test data in a ratio of 80/10/10.

Table 1 shows an overview of the model performance achieved by four ViTMAEs of significantly different sizes and a comparison between training from scratch (no pre-training), linear probing, and full fine-tuning. Pre-training and subsequent fine-tuning significantly improve model performance. The best results are achieved when the entire model is fine-tuned (full-fine-tuning). But, even linear probing achieves higher accuracy for model sizes M and L than training from scratch. It should be noted that linear probing is extremely fast, while the weights of the encoder remain frozen after pre-training.

For the two smaller models, T and S, the increase in performance due to pre-training is significantly lower. This means that a minimum size of the model is required so that it can build up a useful internal representation of ultrasonic signals during pre-training. Model M delivers the best overall result, with 99.02% accuracy distinguishing between the intact and defective rope segments while the rope is moving. A further increase in model size does not result in any further increase in performance and is at the cost of training duration.

Finally, it can be concluded from the results of the two AI approaches that the evaluation of the rope condition works very well with both methods. The classifier of the dense-convolutional parallel neural network had a 100% accuracy in defect detection on bare wire ropes and a 97.2% accuracy on galvanised cables, whereby soiling was also present in the latter tests. This results in an average accuracy of 98.6% for this method. In fact, the cable must at least be stationary for the measurement. A fully dynamic method using a transformer-based method with a ViTMAE architecture in conjunction with a fully fine-tuned downstream model resulted in an accuracy of 99.02% in detecting damage, which is slightly higher than the static method but does not take abrasion and contamination into account.

## 4. Discussion

In this paper, we introduced an approach for the in-process monitoring of steel wire ropes. We conducted different strategies from conventional signal evaluation to deep learning approaches with dense-convolutional parallel neural networks and also transformer-based architectures. While the standard approach initially revealed promising aspects, the method was limited by the fact that the evaluation of different rope segments resulted in an offset of the measured variables, thus making the differentiation between diverse rope conditions complex, especially if disturbances like soiling were present.

The approaches using neural networks, on the other hand, could resolve the differences in the data and provide very accurate predictions not only regarding the rope condition but also for the degree of damage if present. One drawback is certainly the fact that a training dataset is required to adjust the AI models for the respective use case, which means that different types of cables require different training data.

While the first strategy with a parallel neural network including both convolutional and fully connected layers of neurons for the processing of series and scalar data could already differentiate the rope conditions and damage levels even with soiled ropes, it was only able to perform well with stationary recorded data, which limits the use case.

The second, transformer-based, approach showed that a reliable distinction between intact and defective rope segments is possible even with moving ropes. Those models that were pre-trained in advance performed best. Particularly, the two larger models (*M* and *L*) benefit significantly from the prior knowledge gained through pre-training, whereas the smallest model (*T*) benefits less from pre-training. This leads to the conclusion that a minimum model size is necessary to build up an internal abstract representation of the ultrasound signals during pre-training. It is observed that pre-training makes it possible to train transformer models with several million weights without notable overfitting using comparatively few labelled training data.

One disadvantage is that pre-training requires computationally intensive model training with a large volume of data, adding complexity to this approach. However, it is important to note that, aside from achieving better accuracies, once pre-trained, a model can be applied to many different downstream tasks with just simple fine-tuning. Fine-tuning is particularly efficient during linear probing as it involves training only a few weights in the final classification layer. However, the results from linear probing are not as accurate as those achieved with more computationally intensive full fine-tuning.

Overall, the approach of generative pre-training of transformers holds considerable potential for enhancements in the context of ultrasound signals. With appropriate training data, a Foundation Model specialised in ultrasound time signals could be developed. Such a model could be utilised across various applications within the ultrasound domain through simple fine-tuning.

At this point, it is worth mentioning the other approaches for the detection of steel wire rope defects documented in the literature, as well as their achieved accuracy. Li et al. used a neural network in conjunction with an optical measurement system and achieved an accuracy of 93% during the detection of a single broken wire out of 100 wires in the wire rope [44]. Zhang et al. achieved a similar accuracy of 93.75% while identifying two broken wires in a cable with a combined approach of a residual magnetic field and image processing through a neural network [45]. Another study achieved 93% recognition accuracy regarding damaged wires by image processing with an autoencoder approach [46]. While these studies reveal a high accuracy over 90% in wire break detection, all the methods used, at least partially, involve optical sensors that have certain advantages but are not able to measure the defects on the inner layers of the cable. In contrast, the proposed methods in this paper can account for these and, depending on the approach, are capable of also measuring other types of defects like abrasion or enable measuring in a dynamic scenario. Hereby, the methods proposed in this paper achieve a very high accuracy, with 98.6% for the stationary and 99.02% for the dynamic approach. This is the highest detection accuracy including inner damage of the rope to our best knowledge.

A performance test to determine the smallest measurable defect quantity is still pending. It must be verified to what extent the temperature has an influence and how the model can take this into account. The measurement of longer ropes in field operations is also a crucial aspect that must be considered, along with wire ropes under varying tension levels. Given the changes in strain regarding cables, it is anticipated that the ultrasonic propagation characteristics will undergo alterations due to the shifts in the material properties and the altered cross-sections of the wires. This will undoubtedly have a significant impact on future research. We are also considering alternative coil designs that would enable the sensors to be constructed as half-shells in the future. This would overcome the limitation of our current EMAT design, which can only be installed by sliding them over the rope from the end. This currently prevents the installation of sensors on ropes where the ends are not accessible.

Particularly, for the use case of moving rope systems, this method in conjunction with the developed and optimised EMAT sensor can provide significant measurement accuracy and reduce inspection times as well as preventing intact ropes being discarded early.

## Figures and Tables

**Table 1:** Comparison of model performance between training from scratch (no pre-training), linear probing, and full fine-tuning. Four different model sizes were analysed. The different model sizes were created by stepwise increasing the model dimensionality as well as the number of attention heads and layers, both in the encoder (ENC) and in the decoder (DEC). Model size M delivers the best results overall. Significant performance improvements were observed for all models with pre-training followed by fine-tuning.

	Dimensions per Attention Head		Attention Heads per Layer		Number of Layers (Blocks)		Params		Test Accuracy in %		
	ENC	DEC	ENC	DEC	ENC	DEC	ENC	DEC	no pre-training	linear probing	full fine-tuning
Tiny (T)	32	16	1	1	2	1	17 K	265 K	89.86	87.22	**91.53**
Small (S)	64	32	2	2	3	1	150 K	539 K	88.05	86.66	**96.39**
Medium (M) *	128	64	4	4	6	2	1.9 M	1.2 M	85.83	90.56	**99.02**
Large (L)	192	96	6	6	9	3	9.3 M	2.4 M	87.64	89.99	**98.47**

* Model with the best overall performance.

**Figure A1:**
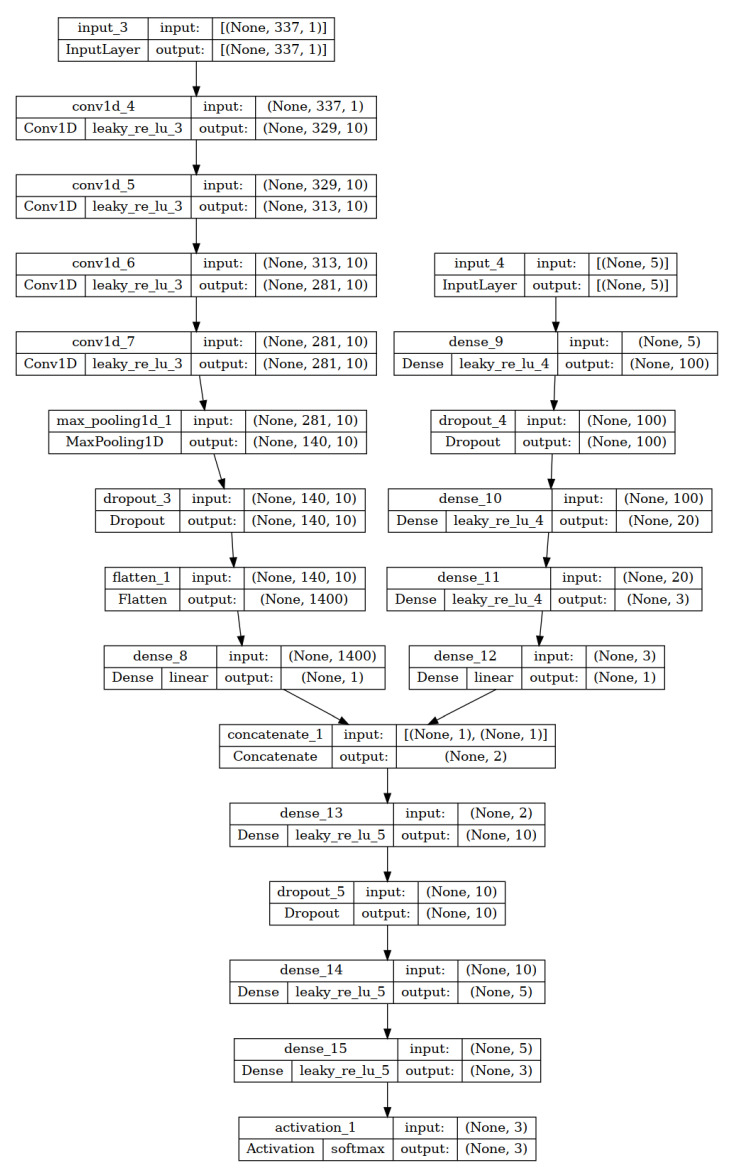
Structure of the classifier neural network for the static case.

**Figure A2:**
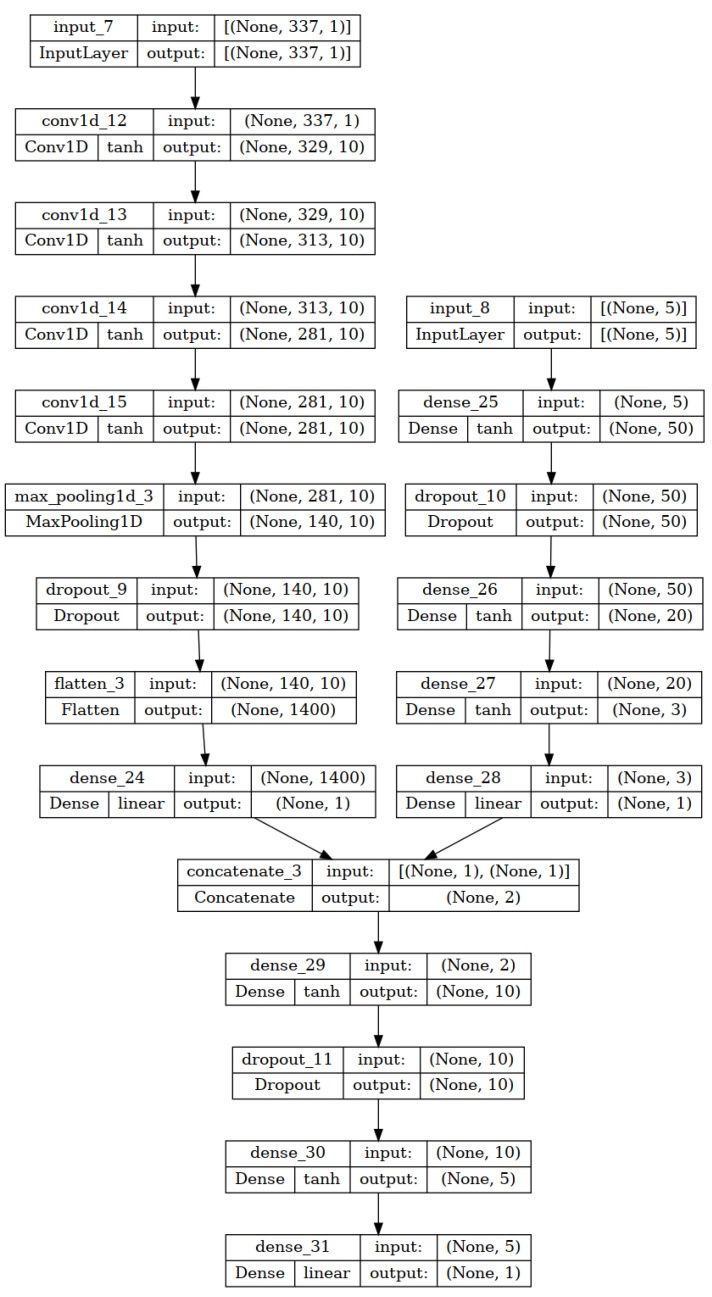
Structure of the regressor neural network for the static case.

## Data Availability

The data that support the findings of this study are available from the corresponding author upon reasonable request.

## Abbreviations

The following abbreviations are used in this manuscript:

EMAT	Electromagnetic Acoustic Transducer
PCA	Principal Component Analysis
AI	Artificial Intelligence
CNN	Convolutional Neural Network
RNN	Recurrent Neural Network
NLP	Natural Language Processing
ViT	Vision Transformer
ViTMAE	Vision Transformer Masked Autoencoder
ToF	Time of Flight
ECN	Time of Encoder
DEC	Time of Decoder
